# Emerging Roles of Long Non-Coding RNAs as Drivers of Brain Evolution

**DOI:** 10.3390/cells8111399

**Published:** 2019-11-06

**Authors:** Geraldine Zimmer-Bensch

**Affiliations:** Institute for Zoology, RWTH Aachen University, Division for Functional Epigenetics, Worringerweg 3, 52074 Aachen, Germany; zimmer@bio2.rwth-aachen.de; Tel.: +49-241-8020844

**Keywords:** lncRNA, translation, transcription, splicing, brain, cerebral cortex, neurogenesis, synaptic plasticity, neurons

## Abstract

Mammalian genomes encode tens of thousands of long-noncoding RNAs (lncRNAs), which are capable of interactions with DNA, RNA and protein molecules, thereby enabling a variety of transcriptional and post-transcriptional regulatory activities. Strikingly, about 40% of lncRNAs are expressed specifically in the brain with precisely regulated temporal and spatial expression patterns. In stark contrast to the highly conserved repertoire of protein-coding genes, thousands of lncRNAs have newly appeared during primate nervous system evolution with hundreds of human-specific lncRNAs. Their evolvable nature and the myriad of potential functions make lncRNAs ideal candidates for drivers of human brain evolution. The human brain displays the largest relative volume of any animal species and the most remarkable cognitive abilities. In addition to brain size, structural reorganization and adaptive changes represent crucial hallmarks of human brain evolution. lncRNAs are increasingly reported to be involved in neurodevelopmental processes suggested to underlie human brain evolution, including proliferation, neurite outgrowth and synaptogenesis, as well as in neuroplasticity. Hence, evolutionary human brain adaptations are proposed to be essentially driven by lncRNAs, which will be discussed in this review.

## 1. Introduction

Recent improvements in advanced sequencing technologies and results obtained from large-scale consortia investigating functional genomic elements like ENCODE and FANTOM [1,2,3] revolutionized our understanding of mammalian genomes in matters of architecture, activity and regulation. In addition to the enormous complexity achieved by protein-coding genes with multiple transcription start sites, alternative promoter and enhancer elements, splicing initiation and donor sites, as well as variable 3′-untranslated regions (UTRs), an unexpected high number of non-coding RNAs (ncRNA) have been identified. Non-coding RNAs are distinguished in small and long non-coding RNAs (scnRNAs and lncRNAs, respectively), which differ in size, biogenesis and function. While most of the sncRNA function refers to posttranscriptional regulation in the cytoplasm [4], the actions of lncRNAs emerged as enormously diverse. The multitude of lncRNA regulatory mechanisms that have been reported so far pervasively influence transcriptional, post-transcriptional and even translational diversification of individual genes as well as whole gene networks [5,6]. Hence, lncRNAs supply neurons with the capacity to very precisely control the spatiotemporal deployment of genes, a prerequisite for the brain’s capability of executing complex neurobiological traits.

In sharp contrast to the highly conserved repertoire of protein-coding genes, thousands of new lncRNAs have appeared during primate nervous system evolution. In the human genome, about 40% of the identified lncRNAs are specifically expressed in the brain [7], referring to 4000–20,000 lncRNA genes. This number is remarkably high considering the approximately 20,000–25,000 protein-coding genes [8] and argues for widespread functional implications. In support of this, lncRNAs show precise regional, cellular and subcellular expression patterns in the brain, which are dynamically remodeled during brain development [9,10,11,12], in response to neuronal activity [13,14,15] and brain aging [16].

Indeed, numerous lncRNAs have been described to be implicated in modulating genes related to neurodevelopment (reviewed in [17]). As studying brain development is appreciated to hold great promise for understanding human brain evolution [18], neurodevelopmental functions of lncRNAs are assumed to be relevant for the evolution of human-specific brain traits [5,17]. Hence, due to their evolvable nature, their specific expression in the brain and their broad functional spectrum, lncRNAs are suggested as crucial drivers of human brain evolution [5,17], which will be discussed in this review. As the cerebral cortex represents the most evolved structure of the human brain and the seat of higher cognitive functions, special focus is laid here on putative lncRNA function in cortical evolution. In that sense, hallmarks of rodent and primate cortical development in the context of suspected evolutionary implications are described comprehensively and comparatively, to highlight the potential lncRNAs have in the light of brain evolution by orchestrating underlying cellular processes.

## 2. Main Text

### 2.1. Biogenesis and Functional Diversity of lncRNAs

lncRNAs are defined as transcripts of at least 200 nucleotides in length. Alike protein coding genes, lncRNAs undergo 5′capping, 3′polyadenylation, splicing modifications and are dependent on their function shuttled to the cytoplasm [19]. They are transcribed by RNA polymerase II from diverse genomic regions, including intergenic regions, introns of protein coding genes as well as in an anti-sense orientation to genes [20,21,22,23,24], from gene regulatory regions including UTRs [25], promoters [26] and enhancers [23], in addition to specific chromosomal regions like telomeres [27].

**Figure 1:**
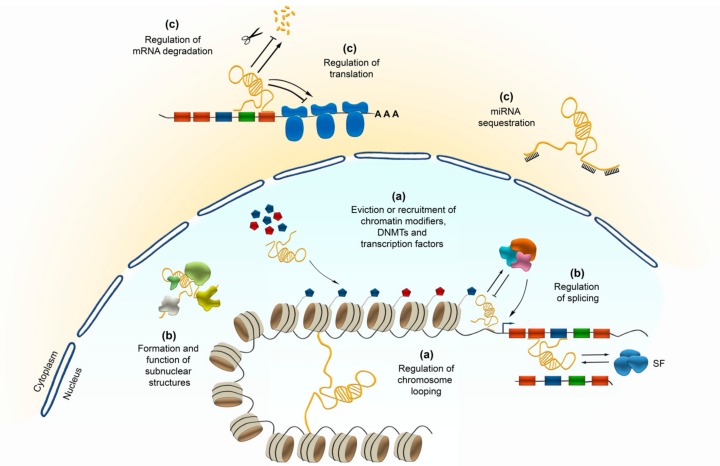
Potential functional diversity of long-noncoding RNAs (lncRNAs) in regulating transcription (**a**), posttranscriptional processes in the nucleus (**b**), as well as potential implications in interfering with translation (**c**) in the cytoplasm.

Apart from their genomic location, lncRNAs can be categorized according to their function. Globally, lncRNAs were reported to be crucially implicated in the regulation of various cellular processes through transcriptional modulation, post-transcriptional control (alternative splicing), nuclear-cytoplasmic shuttling, translational inhibition, mRNA degradation, RNA decoys and regulation of protein activity [28,29] (Figure 1a–c). Beyond that, lncRNAs can also act as precursors for small ncRNAs, such as miRNAs and small nucleolar RNAs (snoRNAs) [5] (Figure 1c). Their functional diversity relies on the inherent properties of RNA molecules, like their modular organization and the ability to fold into different structures. This enables to conduct molecular interactions with other nucleic acids (RNA and DNA), and proteins as well. Dependent on the length of their sequences which can exceed 200 base pairs by far, lncRNAs contain multiple functional domains capable of interacting with different factors coordinating their activity in space and time [6].

### 2.2. Transcriptional Control by lncRNAs

Transcription regulation executed by lncRNAs can be achieved through a broad mechanistical spectrum. Thereby, lncRNAs can act in cis or trans, affecting the transcription of particular local or distal genes, respectively, or even of larger genomic regions like during *XIST*-induced X-chromosome inactivation [30]. lncRNAs can recruit or evict the binding of transcription factors, DNA methyltransferases or chromatin modifiers (Figure 1a). Apart from that, their structural organization allows lncRNAs to act as a scaffold bringing different chromatin-modifying complexes in close proximity (Figure 1a) [31]. These lncRNA-driven interactions essentially contribute to the regulation of temporal and spatial gene expression, which according to the nature of interaction partners yields in selective repression or activation of genes [32].

Different lncRNAs have been reported so far to promote the activation of gene expression by recruiting histone H3K4 methyltransferases, which in turn catalyse the trimethylation at histone 4 lysine 3 residues leading to transcriptional activation [33,34]. In contrast to H3K4me3, polycomb repressive complex 2 (PRC2)-driven trimethylation at H3K27 residues is associated with condensed chromatin and gene silencing [35]. Several lncRNAs have been described to mediate gene silencing in cis or trans by interacting with the PRC2 complex. For example, the lncRNA *HOTAIR*, expressed in antisense from the HOXC locus, interacts with the PRC2 leading to the H3K27me3-mediated gene silencing of the HOXD locus in trans [36]. In addition to the PRC2, *HOTAIR* interacts with LSD1, which is involved in the removal of activating H3K4me3 marks. Hence, by acting as a scaffold *HOTAIR* concertedly promotes chromatin condensation by bringing the two complexes PRC2 and LSD1 into spatial proximity [37].

The PRC2 is a large multiprotein subunit complex of up to 640 kDa in its dimeric state [38], offering diverse binding and interaction sites. Indeed, a multitude of lncRNAs has been identified to bind to the PRC2 across different species and cell types, hence being implicated in PRC2 targeting and recruitment. Among them, *Kcnq1ot1* represents an important example [39]. *Kcnq1ot1* is implicated in genomic imprinting being transcribed from the paternal allele in mice and associated to the silencing of multiple protein-coding genes spreading over a 1-Mb region within the *Kcnq1* domain, which involves H3K27me3 repressive marks [40,41]. Moreover, the lncRNAs *Malat1* [42], sense and antisense transcripts of *H19* [43], *Anril* [44] and *Meg3* [43,45] and sense and antisense transcripts of *Nespas* [43], *Neat1* [46] and *Air* [43] were described to interact with PRC2.

Apart from histone modifying complexes, lncRNAs interact with DNA/RNA binding proteins including transcription factors and DNA methyltransferases like DNMT1 and DNMT3b, thereby evicting or promoting their binding to the DNA [32], and targeting DNA methylation, which in turn often correlates with transcriptional repression [47]. For example, *Dali*, a conserved central nervous system expressing intergenic lncRNA reported to promote neuronal differentiation, interacts with DNMT1 and regulates the DNA methylation status of CpG island-associated promoters in trans [48].

### 2.3. Implications of lncRNAs in Posttranscriptional and Translational Regulation

Due to the length of their sequences, lncRNAs can contain diverse functional domains that enable the interactions with multiple factors, facilitating their implication in a multitude of biological processes. Apart from transcriptional control, lncRNAs are involved in posttranscriptional regulation including alternative splicing and mRNA stability, but also in nuclear-cytoplasmic shuttling and translational control [49,50,51,52], which will be discussed as follows.

Several nuclear-localized lncRNAs were linked to splicing regulation in animals including *NEAT1*, *MALAT1*, *GOMAFU* and *SAF*, all of which are reported to be expressed in the brain [5,17,51]. Some of them seem to be recognized by splicing factors, influencing their activity by either modulating their posttranslational modifications (e.g., phosphorylation), or by regulating interactions with other splicing factors, and/or with protein-coding (pre) mRNAs. A third mechanism through which lncRNAs can be implicated in alternative splicing is through lncRNA-mediated chromatin remodeling [51].

For proper pre-mRNA splicing and the regulation of alternative splicing patterns a continuous phosphorylation/dephosphorylation cycle of serine/arginine-rich (SR) proteins, a conserved family of proteins largely involved in splicing, is required. While hyperphosphorylation of the SR domain influences the binding of SR proteins to the target pre-mRNA, thereby affecting splice site selection, partially dephosphorylated SR proteins support the first steps of the transesterification reactions [53,54,55]. The phosphorylation status further influences the intranuclear trafficking of SR proteins between nuclear speckles, reported as sites for splicing factor storage and modification, and transcription sites [56,57].

*NEAT1* and *MALAT1* were described to regulate the phosphorylation status of splicing factors. By the interaction with the CLK kinase, *NEAT1* modulates the SRp40 phosphorylation status, which regulates the balance of the processing of the PPARy pre-mRNA into the PARy2 mRNA or PPARy1 isoform [58]. *MALAT1*, which also functions as an oncogene transcript involved in diverse cancer types [59,60], was proposed to modulate the phosphorylation status of SR proteins in the nucleus, including the MALAT1-interacting SRSF1 [61]. While phosphorylated SRSF1 is accumulated in nuclear speckles (NS), SRSF1 dephosphorylation is crucial for the export of mRNA-associated proteins and promotes the interaction with cytoplasmic mRNAs, likely affecting translation [62,63].

In cancer cells, *MALAT1* can further disrupt the formation of a splicing modulator complex through hijacking the SFPQ factor (proline- and glutamine-rich SF; or PSF for PTB-associated SF), thereby inhibiting its interaction with the tumour growth factor PTBP2. SFPQ-released PTBP2 then promotes the proliferation of cancer cells [64].

In support of their functions in alternative splicing regulation, *NEAT1* and *MALAT1* are proposed to shape the three-dimensional genome organization, acting as molecular bridges between specific chromosomal locations and nuclear speckles and paraspeckles (reviewed in [51]).

Another lncRNA being implicated in splicing, and in neuronal development [65,66], brain development [67] and post-mitotic neuronal function [67,68] as well, is *GOMAFU*. Its downregulation leads to aberrant alternative splicing patterns, reminiscent of those observed in schizophrenia-associated genes like DISC1 and ERBB4, that both exert key functions in the developing nervous system [13]. As *GOMAFU* was found to be downregulated in post-mortem cortical tissue from the superior temporal gyrus of schizophrenia patients, the aberrant splicing patterns of *DISC1* and *ERBB4* in schizophrenia are suggested to be a consequence of disturbed *GOMAFU* expression. In support of this, *GOMAFU* was found to directly interact with the SFs QUAKING homolog QKI and SRSF1 [13], through which alternative splicing modulation is likely to be achieved. *GOMAFU* is further reported to be recognized by the splicing factor SF1, participating in the early stages of spliceosome assembly [69].

In the cytoplasm, several lncRNAs target mRNA transcripts and modulate mRNA stability (reviewed in [70]). While lncRNAs such as half-STAU1-binding site RNAs (*1/2-sbsRNAs*) and growth arrested DNA-damage inducible gene 7 (*GADD7*) decrease the stability of mRNAs [71,72], others like the antisense transcript for b-secretase 1 (*BACE1-AS*) and the terminal differentiation-induced ncRNA (*TINCR*) promote mRNA stability [73,74].

Gene expression control at a translational level plays a crucial role in neuronal function providing valuable means for the spatiotemporal management of protein dynamics in synapses, most of which are located far away from the neuron´s soma [75]. Translation can be both repressed or promoted by lncRNAs, whereby different mechanisms are described. The antisense lncRNA AS-*Uchl1* targets the *Uchl1* mRNA to active polysomes, thereby promoting cap-independent translation [76], while the *lincRNA-p21* negatively acts on translation of target transcripts, e.g., by inducing ribosome drop-off [77].

Another mechanism through which lncRNAs influence translation is by competing for miRNA binding. This is achieved by so called competing endogenous RNAs, representing lncRNAs that harbour multiple binding sites of identical miRNAs [78]. Through sequestering miRNA species their binding to coding mRNAs is impeded [79,80,81,82,83], diminishing the miRNA-dependent effects on translation (Figure 1c).

Finally, lncRNAs can act on translation by being precursors for small ncRNAs (Figure 1c). About 100 lncRNAs were predicted to encode for miRNAs [84]. A famous example is *H19*, one of the most famous imprinted genes, which is maternally expressed. *H19* is known to regulate placenta growth presumably by repressing the expression of the Insulin like growth factor 2 (*IGF2*) [85]. Apart from that, exon 1 of *H19* gives rise to *miR-675-3p* and *miR-675-5p* [86]. While *miR-675-3p* targets the gene encoding the anti-differentiation transcription factors *Smad1* and *Smad5*, as important components of the bone morphogenetic protein (BMP) pathway, *miR-675-5p* targets the gene coding for the DNA replication initiation factor CDC6 [86]. Hence, by being the precursor of *miR-675-3p* and *miR-675-5p*, *H19* executes a pro-differentiation function in primary myoblasts and regenerating skeletal muscles [86,87].

To summarize, lncRNAs are implicated in the regulation of gene expression and translation at multiple levels, whereby the so-far identified mechanisms are likely far from being complete.

### 2.4. Indications for Potential Implications of lncRNAs in Human Brain Evolution

In contrast to highly conserved lncRNA promoters whose transcription factor-binding sites correlate with their tissue-specific expression patterns [7,46], and their highly conserved splice-junction motifs [88], lncRNA gene bodies display relatively low evolutionary conservation. This apparent absence of sequence conservation does not necessarily imply a lack of crucial biological functions. Indeed, specific lncRNA functions have been preserved [89,90], and several human lncRNAs have been shown to phenotypically rescue depletion of their homologs in zebrafish [90]. The aforementioned studies emphasize the diverse spectrum of actions of lncRNAs, and their biological significance for development and disease-relevant processes. Such functional diversity in addition to their low evolutionary conservation strongly propose lncRNAs as crucial drivers of human brain evolution and the emergence of human specific traits. In support of that, one-third of human lncRNAs seems to be specific to the primate lineage [7] including hundreds of human-specific lncRNAs [91]. This is in stark contrast to the highly conserved repertoire of protein-coding genes, of which with a few exceptions the vast majority of proteins expressed in the nervous system is strongly conserved across diverse mammalian species [92,93,94]. Moreover, numerous lncRNA loci have experienced positive sequence selection during human evolution. Hundreds to thousands of loci have been identified to date being positively selected in humans relative to other mammalian species [93], with about 50 lncRNA loci being positively selected within specific human populations [95]. For example, the positively selected lncRNA *HARF1* is suggested to drive human-specific cortical development, found highly expressed in Cajal Retzius neurons during human embryonic neocortical development at gestational weeks 7–19, when neuronal specification and migration take place [96]. Interestingly, the positively selected regions of its locus are highly conserved in other mammals, for which it is proposed that the positive selection occurred in a functional domain of the lncRNA to drive adaption. In contrast to this, a surprising lack of positive selection in protein-coding genes related to nervous system function in humans relative to primates and rodents has been described [97,98].

Together, their enormously high regulatory potential, their region and stage-specific expression, the positive selection and emergence of new lncRNA species during primate and human evolution make lncRNAs ideal candidates for having acted as essential drivers of human brain evolution and the emergence of human-specific brain features.

### 2.5. Evolutionary Innervations of the Human Brain

Many cognitive features have been postulated to be unique to humans. While the ability to understand others’ inner states and intentions (also referred to as ‘theory of mind’), is not as unique to humans as initially thought [99]; social cognition [100] enabling intensive cooperation including morality [101] and cumulative culture [102] seems to represent a hallmark of human traits. Another unique feature of the human species is language and vocal learning, which has emerged after the split from chimpanzees [103], and which appears to rely on evolved physiological, neurological and cognitive aspects [103,104].

The outstanding cognitive features of the human brain go in line with structural alterations and complexification. These involve a scaling up of brain size and neuronal number, which is the most obvious, best measurable and most studied feature of human brain evolution [105]. The substantial increase in human brain size is mainly due to the tremendous expansion of the neocortex, characterized by new cortical areas, and a strong increase in connectivity [106]. In humans, the neocortex constitutes more than half of the volume of the human brain [107], and a 10-fold rise in human cortical areas is estimated compared to early mammals [108]. Higher order associative cortical areas have tremendously been enlarged in the human cortex [109,110]. The frontal and parietal associate areas were suggested as unique to or highly evolved in primates with the frontal associate (prefrontal) cortex being the largest, occupying the anterior part of the frontal lobe and about one-third of the overall cortical surface [111,112]. This area is regarded as key for highest-order cognitive functions in humans, including language, decision making, social behavior and working memory [112,113,114,115]. Besides, four additional motion-sensitive areas have emerged in the human intraparietal sulcus (IPS) compared to rhesus macaques, which are implicated in the processing of three-dimensional forms in relation to motion [116]. The emergence of these and other posterior parietal areas are proposed to boost the processing of visual and somatosensory information, necessary for complex manipulative abilities that are required for tool manufacture and manipulation [117,118].

The human cortex is further characterized by a relative expansion of the upper cortical layers. Moreover, a greater connectivity between cortical areas and with an expanded thalamus is a hallmark of the human brain. Beyond that, the intrinsic organization of cortical circuitry has been evolutionary adapted to achieve higher cortical function in primates [119,120]. This appears to be attributed to a great extent to the enhanced diversity and function of inhibitory c-aminobutyric acid (GABA) interneurons, as the efficiency of cortical circuitry is highly dependent on interneuron function acting as intrinsic modulators essential for higher order processing [121,122].

Another hallmark of human brain evolution is a highly enlarged subplate layer emerging during development, where the earliest cortical circuits are established from the neurons generated first [107]. To better understand how such large and complex brains may have evolved, investigation of the genetic, molecular and cellular mechanisms of brain development and the comparison between species provides valuable information.

### 2.6. Hallmarks of Cortical Development in View of Potential Implications for Evolution

As the cerebral cortex represents the most evolved structure of the human brain, the following paragraph will focus on important aspects of cortical development in humans, as well as in representative vertebrate species. The six-layered mammalian cerebral cortex is formed in a temporally regulated inside-out fashion. Neurons destined for deep layers are generated first, whereas those born later migrate through the already existing deep layers to form the superficial ones [123]. Hence, neuronal identity correlates with the timing of differentiation, for which the proper balance of progenitor cell proliferation and differentiation is crucial for cell fate regulation and the correct formation of the cerebral cortex. Overproduction of stem cells can lead to megalencephaly, whereas the loss of neuronal stem cells caused by precocious differentiation or increased apoptosis results in microencephaly [124]. The precise orchestration of cell-fate choices underlies the sequential activation of cell type-specific gene regulatory programs in dividing embryonic progenitor cells, which is controlled by lncRNAs along various stages.

**Figure 2:**
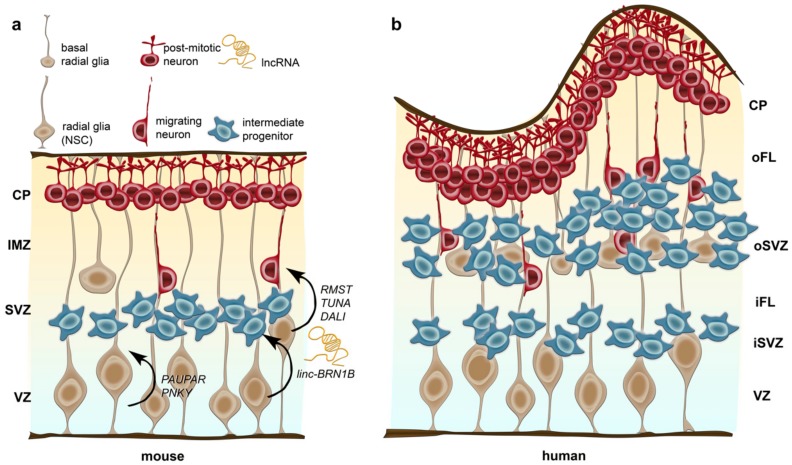
Schematic illustration of neurogenesis in the mouse (**a**) and human cerebral cortex (**b**) and, potential implications of discrete lncRNAs. Radial glia residing in the ventricular zone (VZ) are the neural stem cells (NSCs) in mice and humans, generating neurons, intermediate progenitors and basal radial glia cells. In contrast to intermediate progenitors, radial glia display a long basal process attached to the outer (basal) surface. The subventricular zone (SVZ), which hosts intermediate progenitors and basal radia glia, is dramatically expanded in humans and separated into an inner and outer SVZ (iSVZ and oSVZ, respectively) by the inner fibre layer (iFL). Post-mitotic neurons migrate along the basal processes of the radial glia out of the VZ and SVZ through the intermediate zone (IMZ) in rodents and the inner and outer fibre layer (iFL and oFL) in humans into the cortical plate (CP). In humans the cortex is highly folded in gyri and sulci, whereas the mouse brain is smooth. While *Rmst*, *Tuna* and *Dali* are suggested to drive neuronal differentiation, *Paupar* and *Pnky* appear to be implicated in controlling the balance of self-renewal and neuronal differentiation of neuronal progenitor cells. *linc-Brn1b* controls differentiation of delaminating neural progenitor cells, presumably being involved in basal cortical progenitor turnover regulation.

The human cerebral cortex, which is generated during the first two trimesters of gestation, arises from neuronal stem cells residing in the epithelium of the neural tube (neuroepithelial cells) [109]. These stem cells subsequently produce diverse subtypes of progenitor, neuronal and glial cells. Neuroepithelial cells give rise to radial glial cells (RGCs), which are also called apical progenitors. Apical progenitors reside in the ventricular zone (VZ) and form bipolar radial processes between the ventricular and pial surfaces of the cortex, which serve as scaffold for post-mitotic migrating neurons that form the six-layered cortical structure in an inside-out fashion [125,126,127] (Figure 2).

RGCs can divide symmetrically to expand the pool of progenitor cells [128,129], while asymmetric divisions lead to the generation of post-mitotic neurons, or intermediate, transiently amplifying progenitor cells. These intermediate progenitors (IPCs) translocate their cell bodies more basally, for which they are also named basal progenitors, thereby forming another zone called the subventricular zone (SVZ; Figure 2). In rodents, IPCs divide symmetrically to indirectly generate the majority of neurons destined for all cortical layers [130,131,132]. The presence of this basal precursor pool is a mammalian-specific feature absent in sauropsids (birds and reptiles), which display three layered cortices [127,133,134]. Recent experimental [135,136,137,138] and theoretical [139] evidence emphasizes that the expansion of these basal progenitor cells is sufficient to cause an increase in cortical size and folding, mimicking alterations that have emerged during human brain evolution.

In the primate cortex, an outer subventricular zone (oSVZ) is aroused as a developmental anatomical innovation, to which much of the surface and volume expansion of the cerebral cortex is attributed (Figure 2b, reviewed in [140]). The primate oSVZ is separated from the inner subventricular zone (iSVZ) by a thin layer, which is rich in axonal fibres and known as the inner fibre layer (iFL). The outer boundary of the oSVZ is formed by an outer fibre layer (oFL) [141,142] (Figure 2b). The oSVZ displays particular features very different to the ones of the loosely organized SVZ of rodents and primates [143]. During the time course of primate and human corticogenesis the oSVZ rapidly expands, whereas the VZ (as the major proliferative region in rodents) rapidly declines in macaques and humans [72,142,144,145]. A characteristic feature of the large basal progenitor pool of the pronounced primate oSVZ is the maintenance of radial glial morphology in a large subset of precursors, which are called basal radial glia cells (bRGCs) [144,145,146,147]. While being also identified in mice, only a few (less than 0.5%) bRGCs have been reported for the lissencephalic mouse cortex [148,149], which appears to lack an oSVZ (Figure 2a). In the moderately gyrified cortex of carnivore ferrets, an oSVZ-like layer with bRGCs has been described during development [144,150], although not as pronounced as in primates. Moreover, the neurogenic potential of bRGCs varies between ferrets and primates producing more astrocytes than neurons in ferrets [150].

Experimental manipulation of bRGC abundances in lissencephalic and gyrencephalic animal models affects cortical folding and surface area [135,136,151]. This suggests a correlation between the magnitude of oSVZ proliferation and cortical size as well as degree of gyrification [152]. However, as the cortex of the marmoset, a lissencephalic primate, also displays a pronounced oSVZ, an enlarged oSVZ containing bRGCs could be seen as an evolutionary trend necessary, but not sufficient for the evolution of large gyrencephalic brains [153,154].

Moreover, a higher degree of diversity of bRGC morphotypes has been observed in macaques compared to rodents [146]. While technical considerations hamper the determination of the exact proportions of the bRGC morphotypes [143], basal process-bearing, apical process-bearing, apical and basal (bipolar) process-bearing bRGCs in addition to non-polar basal progenitor cells have been observed in the macaque oSVZ [143]. The evolutionary changes in basal progenitor morphology, particularly the raise in process numbers, is suggested to be associated to the boosted proliferative capacity in humans [155]. Together, this points to parallel increases in morphological diversity and proliferative capacity in brain evolution.

In addition to the bRGCs located in the oSVZ in humans, IPCs generated from bRGCs represent an abundant progenitor population in the human oSVZ. While they are restricted to the VZ and SVZ in the developing rodent cortex, in the developing human cortex they are vastly expanded at much greater distances from the ventricle (Figure 2) [107]. Underneath areas of gyral growth, IPCs are found higher in numbers and occupy a thicker oSVZ than under developing sulci [152]. Hence, in concert with oRGCs, IPCs contribute to the radial expansion and gyrification of the human brain. In support of this, humans with abnormal IPCs due to deficient *TBR2* expression display severe cortical malformations, characterized by microcephaly and defective gyrus formation (polymicrogyria) [156].

IPCs display complex morphologies, being rather multipolar in the SVZ and oSVZ [155,157], while VZ IPCs in mice are characterized by a short bipolar shape with the apical process attached to the ventricle [157,158]. Recently, IPCs were reported to have more processes in humans compared to mice [155], and the process dynamics appear crucial for interactions with the RGCs of the VZ mediated by DLL1 protein-induced Notch signalling [159]. Notch signalling in RGCs, including bRGC in the oSVZ, prevents their premature differentiation [145].

The diversity of the precursor types in the oSVZ allows for manifold interactions with progenitors of the VZ and iSVZ as well as with post-mitotic neurons of the subplate and cortical plate. Moreover, interactions of oSVZ precursors with thalamic axons invading the cortex along the oFL that borders the basal part of the oSVZ are conceivable [143]. Interactions of thalamic axons with cortical progenitors were also described in mice, which, however, referred to interactions with RGCs [160].

The integration of all these microenvironmental signals by oSVZ precursors in the primate cortex might enhance the flexibility of phenotypic fine-tuning during cortical neurogenesis. This might underlie the complexification of the primate cortex, its laminar organization and dense areal microcircuitry establishing characteristic feedback and feedforward pathways in a counter stream configuration [161], pathways not found in rodents [162]. Hence, the primate oSVZ is special in multiple aspects exhibiting striking differences in the basal progenitor pool compared to rodents, and the increase in the diversity of progenitors appears prerequisite for the rise in complexity of the mature human cortex.

### 2.7. Implications of lncRNAs in Processes Potentially Relevant for Human Brain Evolution

A precise spatiotemporal regulation of stem cell proliferation and differentiation underlies the complex process of brain development, and shifts in the proliferative potential appear to contribute to brain size, a crucial paradigm of human brain evolution especially in regard to the cerebral cortex [107,163].

Deciphering the regulatory programs of neurodevelopmental processes like progenitor and neuronal fate specification, but also migration and circuit formation, and comparing between species seems promising for understanding human brain evolution. Current models rely on a limited number of regulators, most of which represent transcription factors, accounting for a limited number of key nodes within a wide and likely more complex regulatory network [164].

The emergence of primate and human-specific lncRNAs in combination with their diversity of actions capable of modulating large gene regulatory networks and post-transcriptional events at multiple levels make them ideal candidates as drivers of human brain complexity and evolution [17]. Compared to protein-coding genes, lncRNAs are expressed at lower levels with higher spatiotemporal, cell type and tissue specificity, which is vigorously regulated during neuronal development [9,10,67,165]. Against this background, numerous studies have investigated the functional implications of lncRNAs in brain development [166].

#### 2.7.1. Cis- and Trans-lncRNA Regulatory Control over Neuronal Differentiation

Neuronal fate regulation depends on the accurate spatiotemporal control over progenitor cell self-renewal and differentiation [167]. lncRNAs control the sequential activation of cell type-specific gene regulatory programs in proliferating stem/progenitor cells that drive the progression from pluripotent cells in the early embryo through to the terminal cell types evident in the mature mammalian brain. Embryonic stem cells (ESCs) were extensively used to investigate the exit from pluripotency to early neural differentiation. A multitude of lncRNAs were identified as necessary for driving neural lineage entry or establishing pluripotency [46,168,169]. While often being controlled by pluripotency transcription factors, such as OCT4, SOX2 and NANOG, lncRNAs in turn exert their regulatory influence by directing transcription factors or chromatin remodelling machineries to specific lineage-specifying genes in cis or trans (Figure 1a). For example, the transcription factor REST induces the expression of the lncRNA *Rmst*, which drives neural differentiation by recruiting the neural transcription factor SOX2 to key neurogenesis-promoting genes, such as *Dlx1*, *Ascl1*, *Hey2* and *Sps* [169], thereby acting in trans. A similar mechanism was described for *Tuna*, regulating pluripotency and neuronal differentiation of ESCs by forming a complex with three RNA-binding proteins, NCL, PTBP1 and hnRNP-K, which then concertedly target and promote the expression of *Nanog*, *Sox2* and *Fgf4* in trans [170].

The lncRNA *Dali* was shown to promote neural differentiation by driving the expression of essential neuronal differentiation gene expression programs in neuroblastoma cells through diverse mechanisms. It promotes the expression of *Pou3f3* in cis, which together with *Dali* forms a trans-acting regulatory complex regulating the expression of neural differentiation genes. Moreover, *Dali* interacts with DNMT1 to inhibit the DNA methylation of CpG island-associated promoters in trans [48]. The lncRNA *Paupar* regulates the expression of the transcription factor *Pax6* in cis [171], known to be crucial for RGC fate [172]. Moreover, *Paupar* modulates the activity of transcriptional regulatory elements of neuro-developmental genes in trans to regulate transcription programs that influence cell-cycle profiles and differentiation of neuroblastoma cells, in part through interactions with the transcription factor PAX6, but also independent of PAX6 [171].

These examples, which mainly refer to in vitro studies, emphasize how complex gene expression programs may be modulated by individual lncRNAs like *Tuna*, *Rmst*, *Dali* and *Paupar*, thereby acting on cell fate choices. Support from in vivo studies underline the relevance of these findings. Genetic disruption of *Evf2*, one of the first nervous system-specific lncRNAs investigated in detail in vivo, disturbs the balance of excitatory and inhibitory neurons in the postnatal hippocampus and dentate gyrus, caused by defects in GABAergic interneuron specification [173]. *Evf2* controls the expression of the interneuron lineage-specific genes *Dlx5*, *Dlx6* and *Gad1* by cis and trans-acting scaffolding mechanisms, through which the transcription factor DLX and the methyl-CpG-binding protein MeCP2 is recruited to regulatory regions [173].

The lncRNA *Pnky*, which is expressed in the nucleus of dividing neural stem cells (NSCs) in the developing mouse and human brain (Figure 2), controls the balance of self-renewal and neuronal differentiation in dividing NSCs through the regulation of a crucial alternative splicing pathway involving an interaction with splicing regulator PTBP1 [174]. In vivo relevance of lncRNAs for the regulation neurodevelopmental processes is further provided by M Sauvageau et al. [175], showing that the intergenic lncRNA *linc-Brn1b* controls differentiation of delaminating neural progenitor cells. By cis-acting mechanisms, *linc-Brn1b* regulates the levels of its neighbouring BRN1 protein-coding gene, presumably involved in basal cortical progenitor turnover regulation [175] (Figure 2).

Together, these and other studies provide a strong body of evidence for a crucial role of lncRNAs in regulating cell-fate choice and stem/progenitor cell turnover during neural development by executing lineage-specific gene expression programs through a broad spectrum of actions (summarized in Table 1). These include transcriptional as well as post-transcriptional mechanisms, which appear highly spatiotemporally coordinated.

#### 2.7.2. lncRNA-Mediated Regulation of Neurite Outgrowth and Synaptogenesis

One feature of human brain evolution is the elaborated connectivity. During brain development this is achieved by mechanisms acting on neurite outgrowth and complexity, as well as the formation of synapses to establish functional connections, which occurs after the termination of neuronal migration from the proliferative niches to respective target regions. Upon being formed, neurons can change their connectivity and the relative strength of each individual synapse in response to changes in activity. This is called neuronal plasticity and represents the basis for learning, memory and cognition [176].

Neurite outgrowth, synaptogenesis as well as synaptic plasticity require complex regulation of gene expression and signal transduction, to which lncRNAs appearently contribute essentially. Emerging evidence suggests that both, nuclear as well as synaptic lncRNAs are implicated herein.

**Figure 3:**
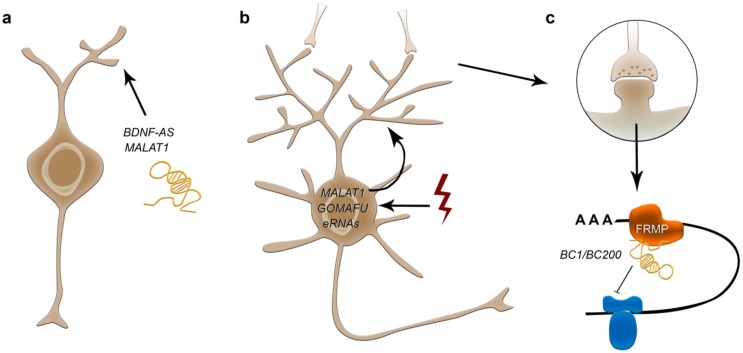
Potential roles of lncRNA in neurite outgrowth (**a**), activity induced synaptic function (**b**) and local translation in synapses (**c**). *Bdnf-AS* and *Malat1* represent important lncRNAs implicated in neurite elaboration (**a**). In addition to enhancer-associated lncRNAs (eRNAs), *Malat1* and *Gomafu* display transcriptional changes in response to depolarization, representing potential candidates to couple neuronal activity to specific posttranscriptional modifications in neuronal plasticity (**b**). *BC1/BC200* is dynamically upregulated at specific synapses in response to neuronal activity being actively trafficked to dendrites, where it controls 48S complex formation and represses local translation in synapses by interaction with FMRP and translational machineries like eIF4a and poly(A)-binding protein (PABP) (**c**).

Antisense lncRNAs were recently described to control the expression of genes implicated in neurite elaboration like *Bdnf*, *EphB2* and *Gdnf* [177]. *Bdnf-AS* lncRNA achieves repression of the BDNF growth factor gene through the recruitment of the PRC2 to the *Bdnf* locus thereby influencing BDNF-mediated effects on neurite outgrowth (Figure 3, Table 1), differentiation, survival and proliferation [177]. Another important lncRNA implicated in neurite elaboration regulation is *Malat1* (Figure 3), which is abundantly expressed in neurons with prominent transcription-dependent enrichment in nuclear speckles, as aforementioned. While *Malat1* knockout mice apparently show no overt phenotype [178], in vitro data using cultured hippocampal neurons point to *Malat1*-dependent regulation of synaptic density [179] (Figure 3, Table 1). By actively recruiting SR-family splicing proteins to transcription sites *Malat1* has been proposed to control the expression of synaptogenesis-related genes [179]. Knockdown of *Malat1* led to decreased synaptic densities, whereas overexpression reciprocally caused an increase [179]. Potential redundancies compensating for the loss of *Malat1* function or very subtle undetected phenotypic effects of *Malat1* knockout mice could explain the evident conflict between the in vivo and in vitro results.

#### 2.7.3. lncRNA-Mediated Regulation of Synaptic Plasticity

Alongside with the reorganization of the brain and the raise in size, neuronal plasticity is proposed to play a major role in explaining the evolutionary history of the human brain, which appears to display more pronounced plasticity compared to our close relatives [180]. Neuronal plasticity relies on the ability to change the set and relative strength of synaptic connections over time in response to sensory experience as well as other environmental cues, and underlies learning, memory and cognition. Hence, neuronal plasticity allows the brain to be moulded by external influences, including the ecological, social and cultural context, and promotes the brain’s capability to recover from injury or insult [180].

While the role of lncRNAs in regulating neuronal plasticity has just begun to be approached, their putative responsiveness to alterations in neuronal activity in combination with their gene regulatory potential make them attractive candidates for being essential regulators in neuronal plasticity, as activity-dependent transcription is key for the process of neuronal plasticity (reviewed in [181]). Activity-dependent transcriptional changes relate the transcriptional output of neurons and hence the protein composition to their recent history of firing, which is required for Hebbian learning.

Among the numerous lncRNAs that were identified as dynamically transcriptionally regulated in response to neuronal activity [13,14,15], enhancer-associated lncRNAs (eRNAs) were found to be rapidly induced upon depolarization in mouse cortical neurons by potassium chloride in vitro (Figure 3). Moreover, their transcriptional changes correlated prominently with alterations in the expression of nearby protein-coding genes [14]. However, the functional consequences for most of these eRNAs that were activity-dependently changed in expression still remain elusive. To follow this line of research is considered to be very promising, as numerous mechanistic studies provide evidence for eRNAs being fundamental for the enhancer function in other biological systems, promoting activity at target genes by recruiting the mediator complex, diverse transcription factors such as CBP, CREB and NPAS4 and RNA polymerase II to enhancer loci [14,182,183,184,185,186,187]. This mechanism is anticipated to occur in the nervous system as well, for which the investigation of activity-dependent eRNAs in neuronal plasticity is considered an exciting future topic [17].

Among the non-enhancer lncRNAs that were identified to be transcriptionally changed in response to depolarization, *Gomafu* and *Malat1* represent potentially interesting candidates [13,15], both of which are abundantly expressed in neurons (Figure 3). As they form ribonucleoprotein complexes within the nucleus that are enriched in splicing proteins, *Gomafu* and *Malat1* are speculated to couple neuronal activity to specific posttranscriptional modifications in neuronal plasticity (Table 1) [17].

High-frequency stimulation-induced long-term potentiation resulted in dynamic changes of lncRNA expression [188], whereby a prominent fraction of these lncRNAs highly correlated with the differential expression of neighbouring protein-coding genes as well as with known LTP genes [188]. These findings strongly implied that lncRNA-dependent transcriptional control is substantially involved in mediating synaptic plasticity.

Apart from their role in transcriptional and post-transcriptional regulation, lncRNAs are known to act on translation, as described previously. Local protein translation is of particular relevance to maintain dendritic and axonal synaptic functional integrity in neurons, as dendrites and axons can extend far from the soma. Translational control at synapses is key for neuronal plasticity regulating long-term changes underlying learning, memory and behaviour (reviewed in [189]). An lncRNA found to modulate translation of specific mRNAs in synapses is *BC1/BC200*, which is expressed in the developing and adult nervous system, and which indeed was the first lncRNA described to affect synaptogenesis regulation [190] (Figure 3). *BC1/BC200* is actively trafficked to dendrites, where it controls 48S complex formation and represses local translation in synapses by interaction with FMRP and translational machineries like eIF4a and poly(A)-binding protein (PABP) [191,192] (Figure 3, Table 1). Through this, *BC1/BC200* acts on spatially restricted synaptic turnover [193,194,195]. Of note, *BC1/BC200* is dynamically upregulated at specific synapses in response to neuronal activity [196]. Hence, *BC1/BC200* can modify synaptic protein composition dependent on local activity and appears therefore central for synaptic plasticity regulation. Detailed behavioral studies with *BC1*-deficient mice support relevance of *BC1/BC200*-dependent translational control in neuronal plasticity, showing that genetic deletion results in abnormal activity and a broad spectrum of behavioral deficits [193,194,195]. However, no prominent morphological defects were observed in brains of *BC1*-null mice [194].

While still awaiting empirical support, lncRNA-mediated boosting of synaptic connectivity, organizations of intra and inter-regional circuits, as well as synaptic plasticity may be associated to the exceptional human cognitive skills.

## 3. Conclusions

The discovery of mammalian, primate and human-specific lncRNAs in combination with their evolvable nature led to the question of their biological meaning in the context of brain evolution. Based on numerous studies providing compelling evidence for the essential role of lncRNAs in neurodevelopmental processes and synaptic plasticity that are assumed to have contributed fundamentally to human-specific brain traits, lncRNAs appear as attractive candidates for drivers of human brain evolution. Apart from their specific spatiotemporal expression patterns, lncRNAs display an enormous functional diversity ranging from transcriptional to post-transcriptional and even translational level. Their modular organization allows not only for a great spectrum of interactions with and scaffolding of RNA, DNA and proteins, but also for the independent generation of new functional properties for each domain and hence, the establishment of novel combinations.

Apart from acting within a given cell, intercellular communication via vesicle-mediated transport of lncRNAs, as well as small ncRNAs and mRNAs, emerge as relevant physiological and developmentary mechanisms [197,198,199] that could influence local postsynaptic properties. Hence, enriching the ways of information in neuronal communication through lncRNAs could have further contributed to the boost in computational power characterizing the human brain. In this context, it is worth mentioning that in contrast to their primary definition of being incapable of encoding polypeptides, recent studies propose a potential of lncRNAs for encoding functional micropeptides (reviewed in [200,201]). Indeed, a few studies confirmed small open reading frames (length < 300 nt) for some lncRNAs that could code for a short peptide with key biological functions, some of which are also implicated in CNS development (reviewed in [200,201]). Short peptides could also be involved in intercellular communication relying on vesicle mediated transport [202], adding another layer of complexity in lncRNA-mediated regulation of neuronal development and communication.

## Figures and Tables

**Table 1:** List of lncRNAs with a putative function in neurogenesis and neuronal circuit formation.

Process	lncRNA	Biological Function/Phenotype	Molecular Function
**Neural stem cell proliferation and differentiation**	*Pnky*	Regulates neuronal differentiation of embryonic and adult NSPCs	*Pnky* together with *PTBP1* regulate the expression and alternative splicing of an overlapping set of transcripts to promote neurogenesis
	*Paupar*	Knockdown of *Paupar* induces neural differentiation of Neuro-2a neuroblastoma cells	*Paupar* regulates *Pax6* expression locally in cis. Trans: *Paupar* also associates with PAX6 protein and localizes at promoters of *Sox2*, *Nanog*, and *Hes1*
	*Rmst*	Promotes neuronal differentiation	*Rmst* interacts with SOX2 to regulate neurogenic genes including *Ascl1* and *Dlx1* in trans
	*Tuna*	Regulates pluripotency and neural differentiation of ESCs	*Tuna* forms a complex with three pluripotency related RNA-binding proteins, PTBP1, hnRNP-K, and NCL
	*linc-Brn1b*	controls differentiation of delaminating neural progenitor cells	Cis regulation of neighbouring BRN1
	*Gomafu*	Controls retinal development; Dysregulated in schizophrenia	*Gomafu* regulates splicing of neuronal genes, including *DISC1, ERRB4*, and *WNT7B*, probably via association with splicing factors SF1, SRSF1, and QKI
	*Dali*	Depletion of *Dali* in Neuro-2a neuroblastoma cell inhibits its neuronal differentiation induced by retinoic acid	Cis: *Dali* maintains *Brn1* expression. Trans: *Dali* interacts with the DNMT1 to regulates DNA methylation status of CpG island-associated promoters; interacts with BRN1 to regulate expression of neural differentiation genes
**Neurite outgrowth and synaptogenesis**	* Bdnf-AS *	Depletion of *Bdnf-AS* promotes neuronal outgrowth and adult neurogenesis	repression of the BDNF growth factor gene through the recruitment of the PRC2 to the *Bdnf* locus
	*BC1/BC200*	Regulates synaptic excitability, turnover and plasticity	represses local translation in synapses by interaction with FMRP and translational machineries like eIF4a and poly(A)-binding protein
	*Malat1*	Promotes dendrite maturation and synaptogenesis in cultured hippocampal neurons	*Malat1* associates with SR family splicing factors to controls expression of synaptic molecules
** Interneurons **	*Evf2*	Ensures proper formation of GABA-dependent neuronal circuitry	*Evf2* associates with DLX1/2 and MECP2 at the regulatory elements in the *Dlx5/6* intergenic region to control *Dlx5*, *Dlx6* and *Gad1* expression
